# The efficacy and safety of radical prostatectomy and radiotherapy in high-risk prostate cancer: a systematic review and meta-analysis

**DOI:** 10.1186/s12957-020-01824-9

**Published:** 2020-02-24

**Authors:** Zhipeng Wang, Yuchao Ni, Junru Chen, Guangxi Sun, Xingming Zhang, Jinge Zhao, Xudong Zhu, Haoran Zhang, Sha Zhu, Jindong Dai, Pengfei Shen, Hao Zeng

**Affiliations:** Department of Urology, Institute of Urology, and National Clinical Research Center for Geriatrics, West China Hospital, Sichuan University, No. 37 Guoxue Xiang, Chengdu, 610041 China

**Keywords:** High-risk prostate cancer, Radical prostatectomy, Radiotherapy

## Abstract

**Background:**

The optimal treatment for patients with high-risk prostate cancer (PCa) remains a debate and selection of patients to receive proper therapy is still an unsettled question. This systematic review was conducted to compare the effectiveness of prostatectomy (RP) and radiotherapy (RT) in patients with high-risk PCa and to select candidates for optimal treatment.

**Methods:**

PubMed, EMBASE, and Cochrane Central Register of Controlled Trials were searched for eligible studies. We extracted hazard ratios (HRs) and 95% confidence interval (CI) of all included studies. The primary outcomes were overall survival (OS) and cancer-specific survival (CSS); the secondary outcomes were biochemical recurrence-free survival (BRFS), metastasis-free survival (MFS) and clinical recurrence-free survival (CRFS). The meta-analysis was performed using Review Manager 5.3. Subgroup analyses were conducted according to Gleason score (GS), T stage and RT types. Quality of life (QoL) was compared with these two treatments.

**Results:**

A total of 25 studies were included in this meta-analysis. Overall, RP showed more survival benefits than RT on CSS (*P* = 0.003) and OS (*P* = 0.002); while RT was associated with better BRFS (*P* = 0.002) and MFS (*P* = 0.004). Subgroup analyses showed RT was associated with similar or even better survival outcomes compared to RP in patients with high GS, high T stage or received external beam radiotherapy plus brachytherapy (EBRT + BT). As for QoL, RP was associated with poorer urinary and sexual function but better performance in the bowel domain.

**Conclusion:**

RP could prolong the survival time of patients with high-risk PCa; however, RT could delay the disease progression, and combined RT (EBRT + BT) even brought preferable CSS and similar OS compared to RP. RT might be the prior choice for patients with high T stage or high GS. RP could lead to poorer urinary and sexual function, while bringing better performance in the bowel domain.

## Background

About 127,106 patients worldwide are diagnosed with prostate cancer (PCa) annually, accounting for 7.1% of all cancers diagnosed [1]; and it is the most common malignant tumor in the USA [2]. Among men diagnosed with PCa, approximately 20%–30% of patients are grouped as high-risk PCa [3], which is more likely to progress and relapse [4]. To date, radiotherapy (RT) plus androgen deprivation therapy (ADT) has still been the standard treatment for high-risk PCa. In several randomized controlled trials (RCT), RT plus ADT showed better survival benefit than single treatment (RT or ADT alone) [5–10]. Although the level of evidence is low, increasing population-based evidence in recent years has suggested that radical prostatectomy (RP) could provide similar or better survival benefit than RT-based systemic therapy [11–15].

Now, both RT and RP are recommended by current guidelines for patients with high-risk PCa [16]. However, as no large RCT has directly compared the two treatments in high-risk PCa settings, the optimal treatment for this population remains a debate, and selection of patients to receive proper therapy is still an unsettled question. Previous meta-analyses have tried to compare the efficacy of RP and RT in patients with high-risk PCa [17, 18]; however, they failed to perform detailed subgroup analyses for patients with high-risk PCa, such as when patients had different levels of Gleason score (GS) and T stage, or when patients received different types of RT. In fact, it is also unclear whether these differences would affect the comparison between RP and RT. Additionally, limited information was provided by these previous meta-analyses due to inappropriate statistical methods and rough analyses.

Thus, with increasing literature on this topic, we updated this systematic review and meta-analysis to compare the effectiveness of RP and RT in patients with high-risk PCa and select candidates for optimal treatment.

## Materials and methods

### Protocol and searching strategy

This meta-analysis was conducted according to the Preferred Reporting Items for Systematic Review and Meta-Analysis (PRISMA) guidelines [19]. A protocol was developed to define the search strategy and the review was registered on the PROSPERO of the Centre for Review and Dissemination (CRD42019132967). EMBASE (1947 to July 2019), PubMed (1966 to July 2019), and the Cochrane Library database (1948 to July 2019) were searched for relevant studies. We also searched relevant journals and reviews for additional articles. Detailed search strategies and keywords can be found in the protocol.

### Inclusion and exclusion criteria

Inclusion criteria included (a) patients with high-risk PCa: National Comprehensive Cancer Network (NCCN) criteria (≥ T3 or GS 8–10 or PSA > 20), D’Amico criteria (≥ T2c or GS 8–10 or PSA > 20) or the other criteria; (b) patients who received RP or RT as primary treatment, and RT including external beam radiotherapy (EBRT), brachytherapy (BT) or combined RT (EBRT+BT); (c) articles which reported survival outcomes or disease control using hazard ratios (HRs) to present the results of comparison, or articles which reported quality of life (QoL); and (d) studies published in English.

Exclusion criteria include (a) patients with metastatic disease; (b) patients with any disease incompatible with the planned treatment; (c) review, editorial or case report; and (d) studies published not in English.

### Study selection and data extraction

Two researchers (W.Z.P. and N.Y.C.) reviewed titles, abstracts, and then full texts to determine the final included studies. The two reviewers independently collected and checked the data from those included studies. For each included study, we extracted information on the first author, publication year, median age, sample size, study design, characteristics of high-risk PCa, comparison of treatments, median follow-up, RT dose, and end-points. Any disagreements or discrepancies were resolved by consultation with a third researcher (C.J.R.).

### Quality assessment and publication bias

Two researchers (W.Z.P. and N.Y.C.) independently evaluated the methodological quality of the included cohort studies according to the Newcastle-Ottawa Scale (NOS) [20]; scores ≥ 7 points were considered as high quality. Patient selection, comparability, and outcomes were assessed to evaluate the quality. RCT was evaluated according to the criteria outlined in chapter 8 of the *Cochrane Handbook for Systematic Reviews of Interventions*. Publication bias was evaluated by a funnel plot.

### Outcomes

The primary outcomes were survival outcomes, including cancer-specific survival (CSS) and overall survival (OS). CSS was defined as the time from RP/RT until death from PCa. OS was defined as the time from RP/RT until death from any cause.

The secondary outcomes were disease control, including biochemical recurrence-free survival (BRFS), metastasis-free survival (MFS), and clinical recurrence-free survival (CRFS). BRFS was defined as the time from RP/RT until biochemical failure. MFS was defined as the time from RP/RT to metastasis. CRFS was defined as the time from RP/RT until metastasis identified via imaging or biopsy-proven local recurrence. And we chose urinary, sexual, and bowel function as the main evaluation indicators of QoL.

### Statistical analysis

The meta-analysis was conducted using Review Manager 5.3 software. The hazard ratio (HR) and corresponding 95% confidence interval (95% CI) were extracted directly from the study reports. If insufficient data were available, supplementary data might be sought directly from the investigators of studies. A fixed-effect model or random-effect model was used for analyses based on heterogeneity among studies. We used the Chi-square and the I-square tests to assess the heterogeneity among the studies. Chi-squared with a *P* < 0.10 or I-square > 50% was considered as significant heterogeneity. Subgroup analyses were performed according to RT types, GS, and clinical T stage. What we need to pay attention to is that we did not conduct a meta-analysis with secondary outcomes in some subgroups since we were unable to extract enough data from these studies.

## Results

### Study and patient characteristics

**Fig. 1:**
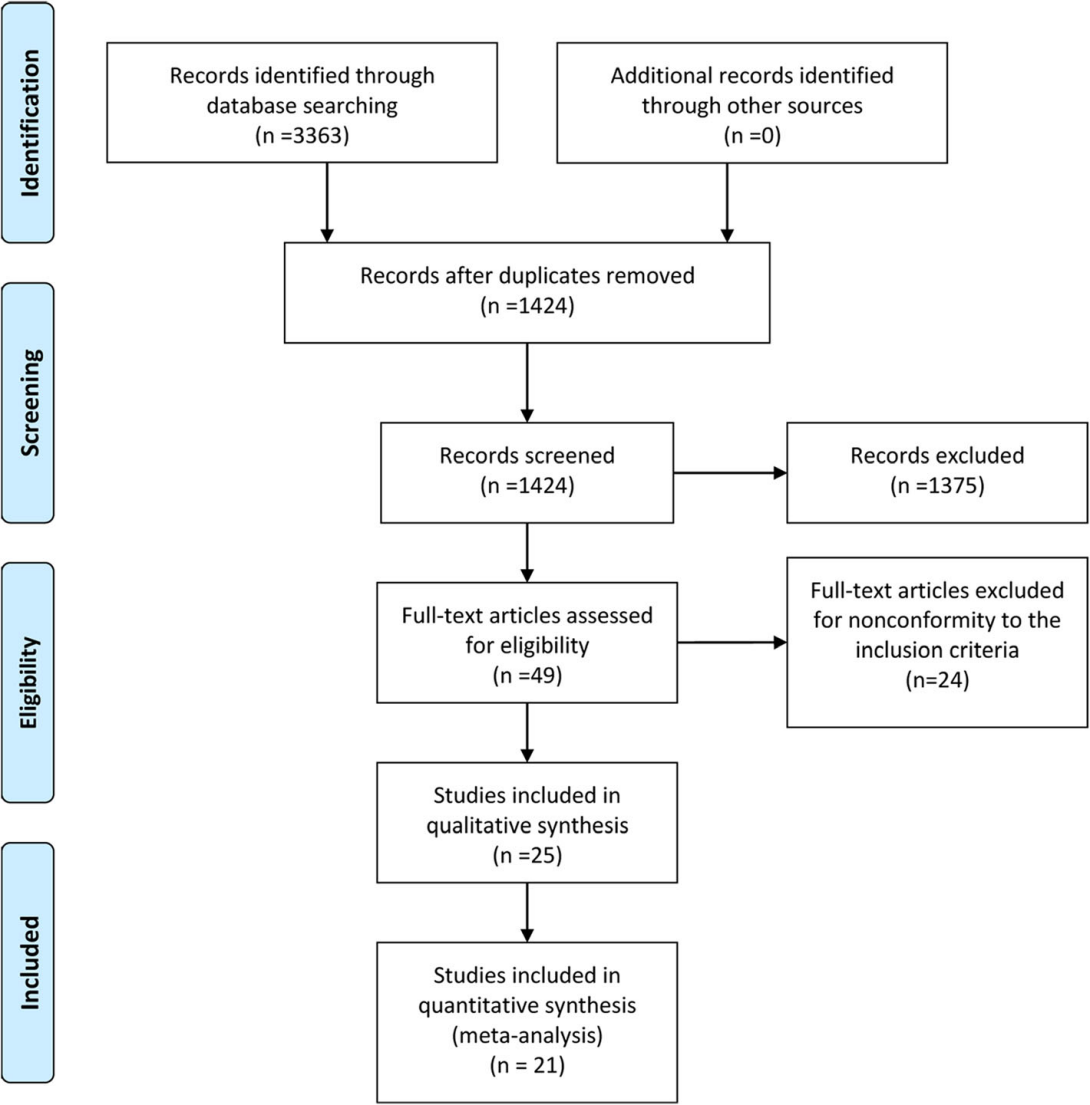
Flowchart of literature searches

Three thousand three hundred sixty three records were identified, and 25 studies were finally included in this systematic review and meta-analysis [11–15, 21–40]. Due to a lack of data, 4 studies cannot be meta-analyzed, so we only presented the results of QoL in these studies. The flowchart of study inclusion was shown in Fig. 1. In total, there were 21 retrospective studies, 3 prospective studies, 1 RCT study published between 2006 and 2019. The median age was 58.2–71.8 years for the RP group and 58.1–75.0 years for the RT group. The median follow-up ranged from 23.89 months to 15 years. The characteristics of all included studies were shown in Table 1. Although the definitions of “high-risk” varied in each trial, most of them were consistent with the NCCN or D’Amico criteria. Detailed comparison data can be found in Additional file 1: Table S1.

**Table 1:** Characteristics of included studies (*N* = 25)

Study ID/date	Study design	Time	Definition of high-risk PCa	Median age (RP vs RT)	Sample (*n*)	Comparison of treatments	Median follow-up	End-points
Jayadevappa 2019	R. cohort	1996–2003	GS ≥ 8 or T ≥ T2c	NA	6296	RP vs EBRT vs EBRT + BT	10 years	OS/CSS
Reichard 2019	R. cohort	2004–2013	NCCN	66 vs 61	304	RP vs RT	82.9 months	OS/MFS
Caño-Velasco 2019	R. cohort	1996–2008	EAU	65 vs 71	286	RP vs EBRT	RP: 152 months RT: 97 months	CSS/OS
Muralidhar 2019	R. cohort	2004–2012	GS: 9–10	NA	4367 2276	RP+aRT vs EBRT+BT	6.0 years (NCDB) 5.8 years (SEER)	OS
Berg 2019	R. cohort	2004–2015	NCCN	58.15 vs 58.12	13985	RP vs EBRT+BT	RP: 91 months EBRT+BT: 101 months	OS
Tilki 2019	R. cohort	1992–2013	GS: 9–10	MaxRT: 70.34 RP: 66. 40 RP+aRT:66.64 MaxRP: 66.04 RP+ADT: 66.38	639	RP vs RP+aRT vs MaxRP vs RP+ADT vs MaxRT	MaxRT: 5.51 years RP: 4.89 years RP + aRT: 3.87 years MaxRP: 4.88 years RP + ADT: 4.65 years	CSS/OS
Jang 2018	R. cohort	1992–2009	T ≥ T3	NA	13856	RP+aRT vs RT	14.6 years	CSS/OS
Tyson 2018	P. cohort	2011–2012	D’Amico	65	2117	RP vs EBRT	3 years	QoL
Ennis 2018	R. cohort	2004–2013	NCCN	RP vs EBRT vs EBRT+BT: 62.61 vs 69.66 vs 67.15	42765	RP vs EBRT vs EBRT+BT	36.34 months	OS
Gu 2018	R. cohort	2004–2008	NCCN	66	7656	RP vs EBRT	NA	CSS/OS
Markovina 2018	R. cohort	2002–2011	NCCN	62.9 vs 64.2	246	RP vs EBRT	RP: 41 months RT: 51.4 months	OS/MFS
Kishan 2018	R. cohort	2000–2013	GS: 9–10	RP vs EBRT vs EBRT+BT: 61 vs 67.7 vs 67.5	1809	RP vs EBRT vs EBRT+BT 61 vs 67.7 vs 67.5	RP: 4.2 years EBRT: 5.1 years EBRT + BT: 6.3 years	CSS/OS/MFS
Robinson 2018	R. cohort	1998–2012	NCCN	63.1 vs 67	41953	RP vs RT	RP: 7.3 years RT: 6.9 years	CSS
Feldman 2017	R. cohort	1992–2009	T3	71.76 vs 71.75	2935	RP vs EBRT	RP: 11.47 years EBRT: 7.04 years	OS/CSS/QoL
Ciezki 2017	P. cohort	1996–2012	NCCN	LDRBT vs EBRT vs RP: 70 vs 68.5 vs 62	2557	LDRBT vs EBRT vs RP	LDRBT: 48.9 months EBRT: 94.6 months RP: 55.6 months	BRFS/CSS/QoL
Yamamoto 2015	P. cohort	2006–2010	NCCN	67 vs 71	150	RP vs EBRT	2 years	QoL
Sun 2014	R. cohort	1992–2005	T2c	70 vs 73	67087	RP vs RT vs WW	NA	OS/CSS
Hoffman 2013	R. cohort	1994–1995	PSA > 10 or GS 8–10	NA	1655	RP vs EBRT	15 years	OS/CSS
Kibel 2012	R. cohort	1995–2005	D’Amico	RP vs EBRT vs BT: 60 vs 69 vs 68(Clinic 1) 61 vs 70 vs 69(Clinic 2)	10429	RP vs EBRT vs BT	67 months	OS/CSS
Westover 2012	R. cohort	1988–2009	D’Amico	65 vs 70	657	RP vs EBRT+BT	RP: 7.6 years EBRT + BT: 3.6 years	CSS
Boorjian 2011	R. cohort	1988–2004	NCCN	RP: 66 EBRT+ADT: 68.8 EBRT: 69.3	1847	RP vs EBRT+ADT vs EBRT	RP: 10.2 years EBRT + ADT: 6.0 years EBRT: 7.3 years	OS/CSS
Aizer 2009	R. cohort	1997–2005	MSK/NCCN	NA	556	RP vs EBRT	RP: 46 months EBRT: 40 months	BRFS
Takizawa 2009	R. cohort	1998–2004	NCCN	64.9 vs 71.1	162	RP vs EBRT	41 months	OS/BRFS/QoL
Arcangeli 2009	R. cohort	2003–2007	NCCN	65.5 vs 75	284	RP vs EBRT	RP: 33.8 months EBRT: 38.6 months	BRFS
Akakura 2006	RCT	1989–1993	T2b-3N0M0	68.1 vs 68.7	95	RP vs EBRT	102 months	BRFS/CSS/OS/QoL

*RP* prostatectomy, *RT* radiotherapy, *EBRT* external beam radiation therapy, *BT* brachytherapy, *ADT* androgen deprivation therapy, *aRT* adjuvant radiotherapy, *WW* watchful waiting, *MaxRP* RP + aRT + ADT, *MaxRT* EBRT + BT + ADT, *LDRBT* low-dose-rate brachytherapy, *PSA* prostate-specific antigen, *GS* Gleason score, *NCCN* National Comprehensive Cancer Network, *MSK* Memorial Sloan Kettering, *QoL* quality of life, *OS* overall survival, *CSS* cancer-specific survival, *BRFS* biochemical recurrence-free survival, *MFS* metastasis-free survival, *CRF* clinical recurrence-free survival, *R. cohort* retrospective cohort, *P. cohort* prospective cohort, *P. and R. cohort* prospective and retrospective cohort, *RCT* randomized controlled trial, *vs* versus, *NA* not available

### Quality assessment and publication bias

Quality assessment was shown in Additional file 2: Table S2. The only 1 RCT was evaluated as high-risk of bias. Twenty three cohort studies were evaluated as high quality (scores 7–9) and 1 cohort study was evaluated as median quality (score 6). A funnel plot was used to evaluate publication bias. As shown in Additional file 3: Figure S1, relative symmetry could be found in the plot, which indicated that there was no obvious publication bias.

### Effect of RP versus RT in all patients with high-risk PCa

**Fig. 2:**
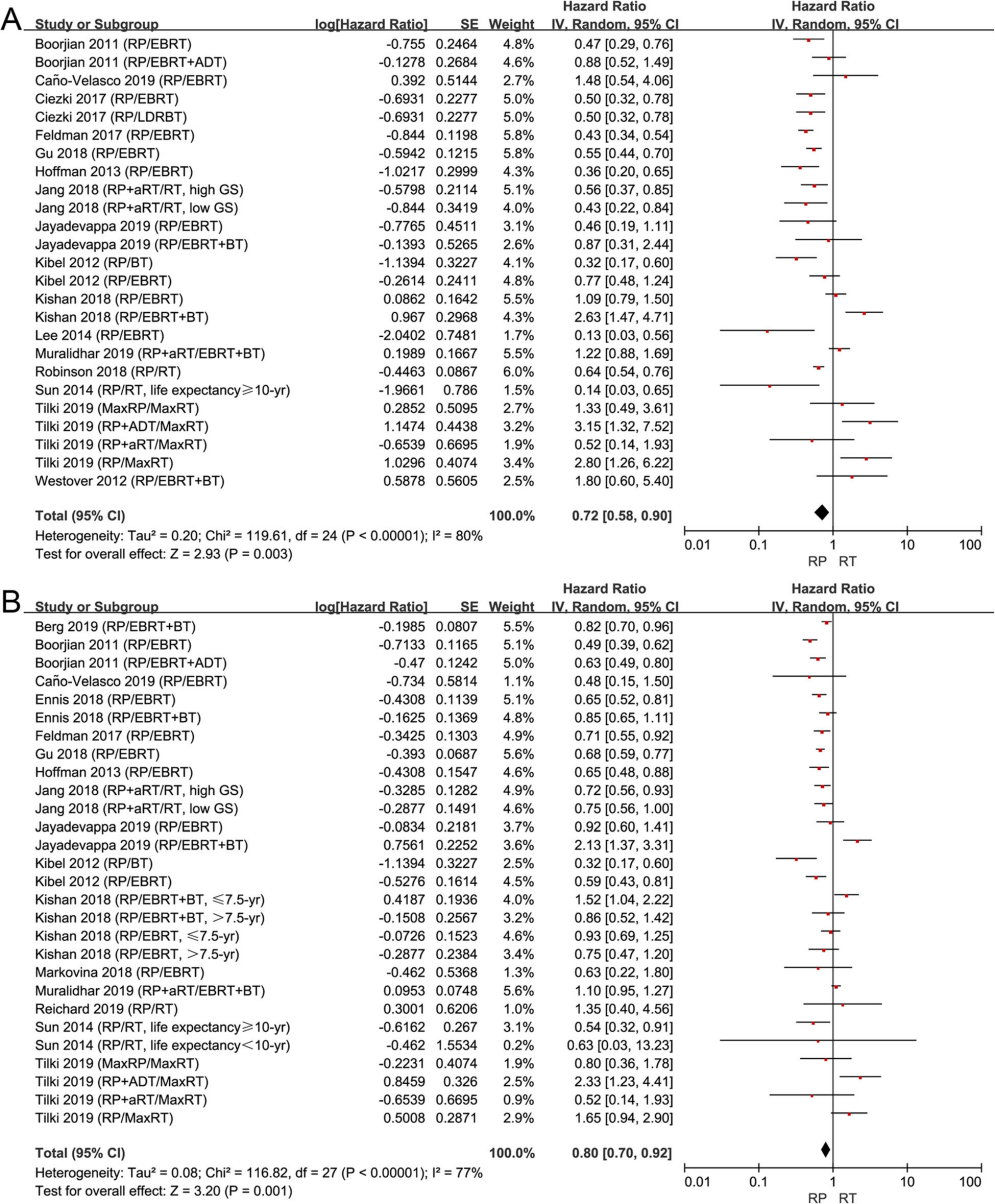
**a** Forest plot of HR for CSS following RP and RT; (**b**) forest plot of HR for OS following RP and RT

From our results, HRs of CSS and OS were reported in 16 studies, respectively. RP showed more survival benefits than RT on CSS (HR 0.72, 95% CI 0.58–0.90, *P* = 0.003, I^2^ = 80%; Fig. 2a) and OS (HR 0.80, 95% CI 0.70–0.92, *P* = 0.002, I^2^ = 77%; Fig. 2b) for patients with high-risk PCa.

**Fig. 3:**
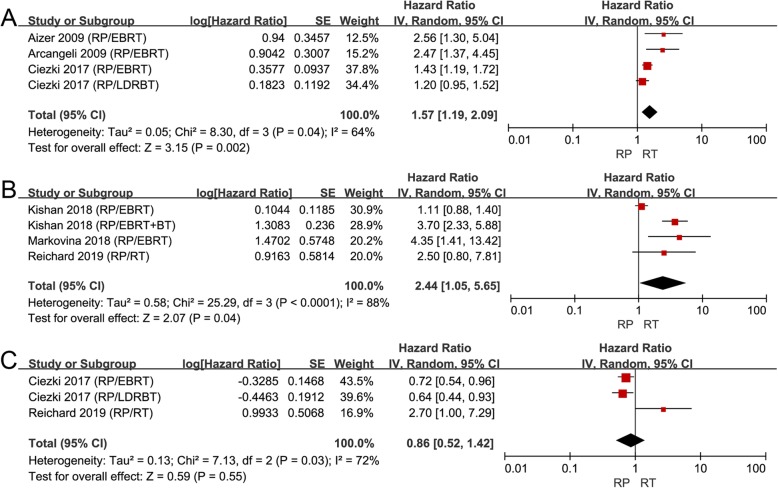
**a** Forest plot of HR for BRFS following RP and RT; (**b**) forest plot of HR for MFS following RP and RT; (**c**) forest plot of HR for CRFS following RP and RT

Although no significant difference was found between RP and RT on CRFS (HR 0.86, 95% CI 0.52–1.42, *P* = 0.55, I^2^= 72%; Fig. 3c), patients treated with RP had a worse outcome of BRFS (HR 1.57, 95% CI 1.19–2.09, *P* = 0.002, I^2^ = 64%; Fig. 3a) and MFS (HR 2.44, 95% CI 1.05–5.65, *P* = 0.04, I^2^ = 88%; Fig. 3b). Taken together, these results suggested that RT could bring better biochemical and metastasis control than RP, although RP could prolong the OS and CSS of these patients.

### Effect of RP versus RT in high GS subgroup

**Fig. 4:**
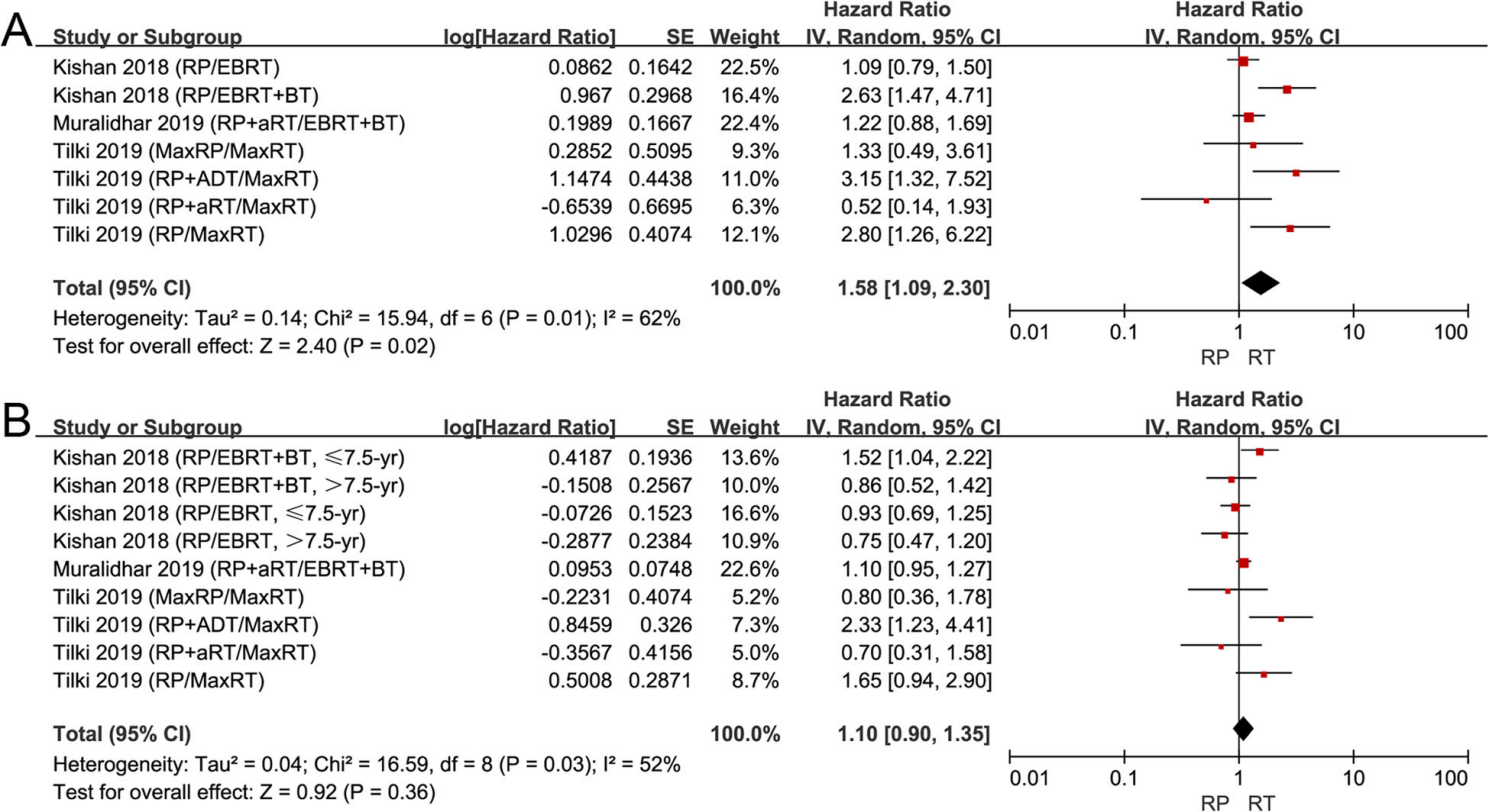
**a** Forest plot of HR for CSS following RP and RT in the “high GS” subgroup; (**b**) Forest plot of HR for OS following RP and RT in the “high GS” subgroup. High GS was defined as GS: 9-10

Some studies reported that PCa patients with GS 9–10 had a particularly aggressive disease [41, 42]. So, we conducted a subgroup analysis for patients with high GS 9–10. Only 3 articles separately compared RP to RT for patients with high GS 9–10 [34, 36, 40]. RT was associated with improved CSS (HR 1.58, 95% CI 1.09–2.30, *P* = 0.02, I^2^ = 62%; Fig. 4a) and similar OS (HR 1.10, 95% CI 0.90–1.35, *P* = 0.36, I^2^ = 52%; Fig. 4b) compared to RP in patients with high GS. RT seemed to have similar or even better survival benefit than RP for these patients. As for other outcomes, only Kishan and colleagues reported that no significant difference was found between RP and EBRT on MFS, while EBRT + BT was associated with longer time to distant metastasis compared to RP [36].

### Effect of RP versus RT in different T stage subgroups

Subgroup analysis was conducted according to the T stage. Due to data limitations, we cannot directly compare the data with complete high T stage and data with complete low T stage. We can only separate two relatively high and low T stage subgroups according to the ratio of different T stages. Then we selected 60% as the cut-off point based on the characteristics of the included studies. Low T stage subgroup was defined as studies that included > 60% patients with ≤ T2 stage and high T stage subgroup was defined as studies that included < 60% patients with ≤ T2 stage.

**Fig. 5:**
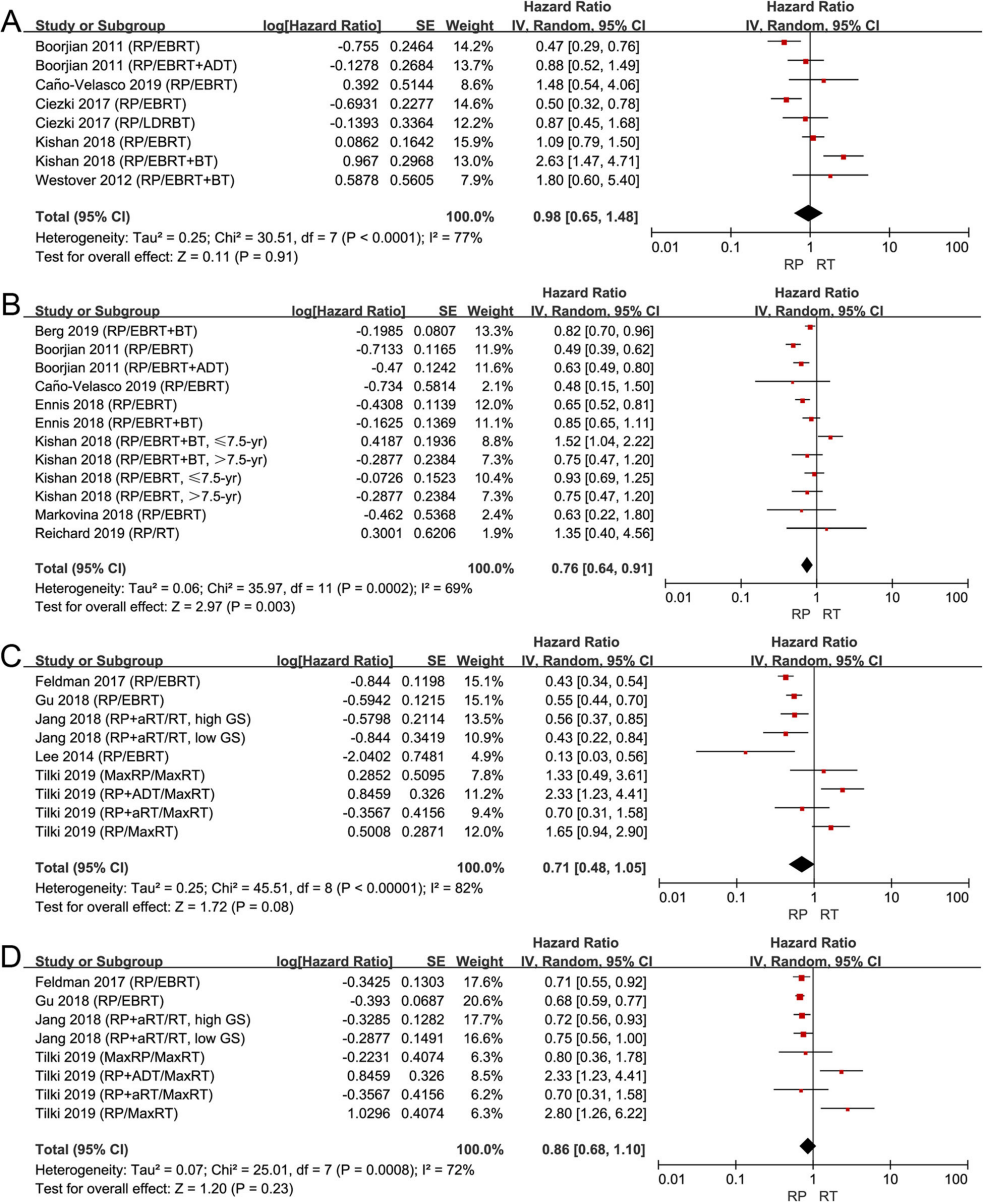
**a** Forest plot of HR for CSS following RP and RT in the “low T stage” subgroup; (**b**) forest plot of HR for CSS following RP and RT in the “low T stage” subgroup; (**c**) forest plot of HR for CSS following RP and RT in “high T stage” subgroup; (**d**) forest plot of HR for OS following RP and RT in the “high T stage” subgroup. “Low T stage” group was defined as studies that included >60% patients with ≤T2 stage; “high T stage” group was defined as studies that included <60% patients with ≤T2 stage

Finally, 9 and 5 studies were respectively grouped into the “low T stage” subgroup and "high T stage” subgroup. In the “low T stage” subgroup, no significant difference was found among patients treated with RP or RT on CSS (Fig. 5a), while RP did extend the OS (HR 0.76, 95% CI 0.64–0.91, *P* = 0.003, I^2^ = 69%; Fig. 5b). However, we found that RT brought a similar survival benefit compared to RP on CSS (Fig. 5c) and OS (Fig. 5d) in the “high T stage” subgroup. With the increase in the T stage, it seemed that RT had better survival benefits.

### Subgroup analysis according to RT types

Patients might receive different types of RT (EBRT or EBRT+BT) in different centers, so we performed a subgroup analysis according to the types of RT. Since the types of RT were not described in detail in some studies, we only included these studies which exactly reported that patients received EBRT or EBRT+BT in different subgroups.

**Fig. 6:**
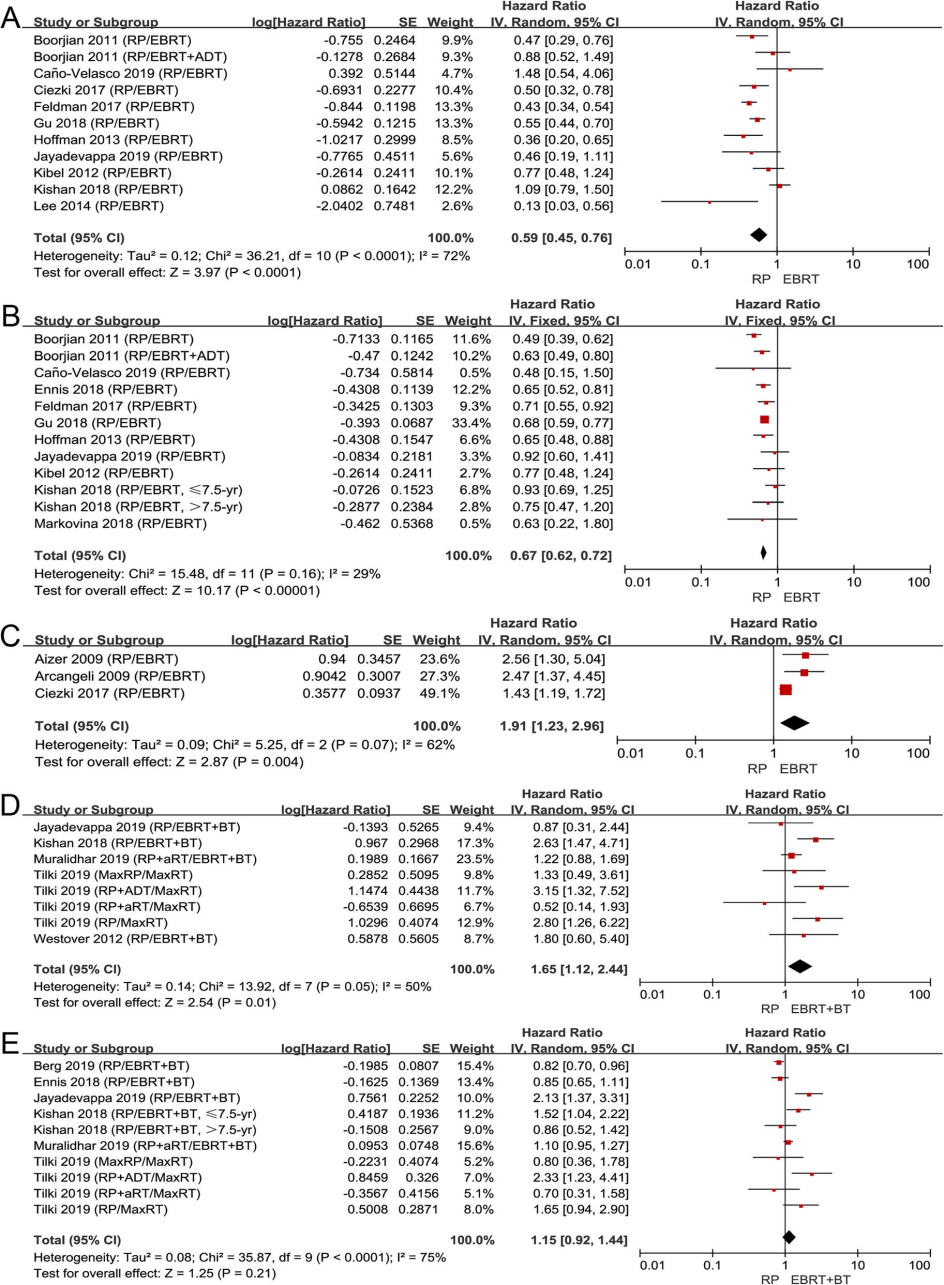
**a** Forest plot of HR for CSS following RP and EBRT; (**b**) forest plot of HR for OS following RP and EBRT; (**c**) forest plot of HR for BRFS following RP and EBRT; (**d**) forest plot of HR for CSS following RP and EBRT+BT; (**e**) forest plot of HR for OS following RP and EBRT+BT

There were 14 articles comparing RP to EBRT and 7 articles comparing RP to EBRT + BT. We separately analyzed the data comparing patients who received EBRT or EBRT + BT to those who received RP. Patients treated with RP had better survival outcomes than EBRT on CSS (HR 0.59, 95% CI 0.45–0.76, *P* < 0.0001, I^2^ = 72%; Fig. 6a) and OS (HR 0.67, 95% CI 0.62–0.72, *P* < 0.00001, I^2^ = 29%; Fig. 6b). Although RP brought better survival benefits, EBRT was associated with better biochemical control than RP (HR 1.91, 95% CI 1.23–2.96, *P* = 0.004, I^2^ = 62%; Fig. 6c), which was consistent with the overall comparison between RP and RT.

It seemed that EBRT+BT had similar or even better survival benefit than RP. RP showed obvious inferiority on CSS (HR 1.65, 95% CI 1.12–2.44, *P* = 0.01, I^2^ = 50%; Fig. 6d) compared to EBRT + BT, and no significant difference between the two subgroups was observed on OS (HR 1.15, 95% CI 0.92–1.44, *P* = 0.21, I^2^ = 75%; Fig. 6e). As for other outcomes, Kishan and colleagues reported EBRT + BT was associated with longer MFS than RP [36].

Overall, most patients received EBRT in the included studies. Although RP was more beneficial to survival than EBRT, it was not as good as EBRT in PSA control, and the survival benefit of RP would no longer exist once EBRT was combined with BT.

### Health-related quality of life

Owing to the limited information available from studies, meta-analysis about the QoL of high-risk PCa patients who received RP/RT was not performed. The QoL was described in 6 studies. Additional file 2: Table S2 showed the characteristics of the included studies which reported QoL. Five studies demonstrated that RT performed better in urinary function. Only Takizawa reported there was no difference between RP and RT on urinary function in the high-risk group (*P* = 0.05) [23]. Three studies showed patients treated with RT had better sexual function than those treated with RP. Two studies reported no significant difference between RP and RT groups about sexual function. Four studies reported no difference between RP and RT on bowel function; the other 2 studies reported that patients in the RP group had a significantly lower risk of suffering from bowel toxicities. It seemed that RP had better performance on the bowel domain, while RT was associated with better QoL in urinary and sexual domains.

## Discussion

Currently, both RT and RP are first-line treatments for clinically high-risk PCa patients, and the optimal treatment remains a debate. One small RCT has compared the survival outcomes for patients with T2b-3N0M0 PCa treated with surgery or radiotherapy [21]. Except for 2 reviews and meta-analysis focused on localized PCa [43, 44], there were two meta-analyses about high-risk prostate cancer published in 2014 and 2015 [17, 18], while limited information was provided due to inappropriate statistical methods and rough analyses. Petrelli and colleagues reported a meta-analysis comparing the efficacy of RP and RT in patients with high-risk PCa and demonstrated the superiority of RP [17]. However, Petrelli and colleagues used odds ratios to present the comparison results, which inevitably ignored the time-to-event outcomes. More recently, a meta-analysis was conducted by Lei and colleagues; they reported that RP brought lower CSM than RT [18], while this meta-analysis was conducted only based on 3 studies. It was particularly noteworthy that none of these previous meta-analyses did a subgroup analysis according to T stage, GS, or RT types, and thus, no detailed data were available for clinicians to optimize treatment strategies.

In the present study, the most up-to-date data were comprehensively analyzed and we found better survival outcomes for patients treated with RP compared to those who received RT. However, RT was associated with better disease control. Subgroup analyses furtherly showed that similar or even better survival outcomes were associated with RT in patients with high GS, high T stage, or received EBRT + BT.

The better disease control for patients treated with RT was likely due to the wider scope of radiotherapy than that of surgery, which made it possible to eliminate micro-metastases outside the prostate and resulted in improved BRFS and MFS. Moreover, adjuvant ADT in addition to RT could further help control the micro-metastases and delay biochemical relapse. However, the improvement of BRFS and MFS by RT did not convert into superior survival benefit compared to RP. Several potential reasons might explain this phenomenon. First, patients treated with RT were older and harbored more adverse clinicopathological features than those with RP. Thus, it was not surprising that patients with RT had a worse prognosis than men with RP. Secondly, to a large extent, the efficacy of RT was determined by the type and dosage. The modality of RT varied in the included studies and the dosage of RT in several studies was lower than what was recommended by current guidelines. Then, patients could choose salvage RT after receiving RP firstly but those patients who chose RT firstly rarely received salvage RP. Last, RT and ADT have greater toxicity than RP, which might lead to the worse OS.

According to our analysis, the types of RT could affect the survival and progression of patients. In fact, several RCT demonstrated a BRFS benefit to EBRT + BT over EBRT [45–47] and several retrospective studies have reported that EBRT + BT brought better outcome than RP on BRFS [39, 48, 49] and MFS [35, 36, 38, 50]. It was not difficult to find that EBRT + BT had strong control over disease progression, which might lead to better CSS for patients treated with EBRT + BT than patients treated with RP. Although EBRT+BT had a better benefit of CSS than RP, this benefit might be neutralized by the increasing other-causes mortality caused by radiotherapy. From our data, we should believe that both RP and EBRT + BT were prior choice than EBRT for patients with high-risk PCa.

As is known, GS is one of the most important prognostic factors [51] and some researches had shown that patients with GS 9–10 had a particularly aggressive disease [41, 42]. Patients with GS 9–10 were at higher risk of disease progression. As mentioned above, RT might have superiority over RP on eliminating micro-metastases and ultimately resulted in better disease control. So these results might be combined to explain the improvement of CSS in patients treated with RT. Similar outcomes were also observed in subgroup analyses according to the T stage. Due to data limitations, we can only use ratios to separate two relatively high and low T stage groups. While we might infer that RT might bring better survival benefits than RP in patients with higher T stage. The inspiration for our clinicians was that RP might be less appropriate and RT might be the first choice for patients with high T stage or high GS.

With the development of treatment modalities, more patients with high-risk PCa can maintain a stable condition for a long term or even be cured. However, making an optimal treatment decision is not only pursuing maximal survival benefit but also a better health-related quality of life. Thus, the assessment of treatments on QoL is also critical for decision making. RP had better performance when considering QoL in the bowel domain, while RT was associated with better QoL in urinary and sexual domains. In clinical practice, younger patients with high-risk prostate cancer who have a greater need for retention of sexual and urinary function after treatment could be recommended with RT. Moreover, RP might be more suitable for patients who need a better bowel function.

Although our research was the most up to date and we did a lot of subgroup analysis, this study still had some limitations. First, heterogeneity was relatively high due to inconsistent inclusion criteria and different treatment modalities. Second, because of the limited data, the population ratio was used to subdivide studies for analysis. Third, in some subgroups, the number of studies and patients is relatively small. Fourth, we failed to perform a meta-analysis on QoL and only presented the results of these studies. This area requires more research and data. Finally, some people included in the studies would inevitably be partially duplicated as the same database was used. Therefore, those results obtained in these subgroups cannot be strong evidence, but can only be used as a reference for interpreting the results.

## Conclusions

In conclusion, RP could prolong the survival time of patients with high-risk PCa; however, RT could delay disease progression, and combined RT (EBRT + BT) even brought similar OS and better CSS than RP. RT might be the prior choice for patients with high T stage or high GS. RP could lead to poorer urinary and sexual function, while bringing better performance in the bowel domain. For clinicians, we should fully consider the patient's characteristics and balance the effectiveness and safety of different treatments when making decisions.

## Supplementary information

**Additional file 1: Table S1**. Results of high-risk group of included studies (*N* = 25).

**Additional file 2: Table S2**. Quality assessment of the included studies.

**Additional file 3: Figure S1**. Funnel plot of radical prostatectomy versus radiotherapy using outcome of overall survival.

## Data Availability

The studies included were all retrieved from PubMed, EMBASE, and Cochrane databases.

## Abbreviations

ADT: Androgen deprivation therapy
aRT: Adjuvant radiotherapy
BRFS: Biochemical recurrence-free survival
BT: Brachytherapy
CI: Confidence interval
CRFS: Clinical recurrence-free survival
CSS: Cancer-specific survival
EBRT: External beam radiation therapy
GS: Gleason score
HRs: Hazard ratios
MFS: Metastasis-free survival
NCCN: National Comprehensive Cancer Network
NOS: Newcastle-Ottawa Scale
OS: Overall survival
PCa: Prostate cancer
PSA: Prostate-specific antigen
QoL: Quality of life
RCT: Randomized controlled trial
RP: Prostatectomy
RT: Radiotherapy
