# Supplementary material for: The efficacy and safety of radical prostatectomy and radiotherapy in high-risk prostate cancer: a systematic review and meta-analysis
Source: World J Surg Oncol. 2020 Feb 24;18:42. doi: 10.1186/s12957-020-01824-9 (PMC7041271; doi:10.1186/s12957-020-01824-9)
Supplement: Supplementary file 1 — Additional file 1: Table S1. Results of high-risk group of included studies (N = 25). [file 12957_2020_1824_MOESM1_ESM.docx]

Supplementary Table 1. Results of high-risk group of included studies (N=25)

| Study ID/date | Comparison of therapies | RT regimen | End points | | |
| --- | --- | --- | --- | --- | --- |
| Jayadevappa 2019 | RP vs EBRT: 677 vs 4141  RP vs EBRT+BT: 677 vs 1478 | NA | CSS | HR (RP/EBRT): 0.46 (0.19-1.11)  HR (RP/EBRT+BT): 0.87 (0.31-2.44) | |
|  |  |  | OS | HR (RP/EBRT): 0.92 (0.60-1.41)  HR (RP/EBRT+BT): 2.13 (1.37-3.31) | |
| Reichard 2019 | RP vs RT: 231 vs 73 | ≥75 Gy | OS | HR (RP/RT): 1.35 (0.4-4.8) P=0.6 | |
|  |  |  | MFS | HR (RP/RT): 2.5 (0.8-7.8) P=0.105 | |
| Caño-Velasco 2019 | RP vs EBRT: 145 vs 141 | 74 Gy | CSS | HR (RP/EBRT): 1.48 (0.54-4.04) P=0.44 | |
|  |  |  | OS | HR (RP/EBRT): 0.48 (0.48-1.50) P=0.57 | |
| Muralidhar 2019 | RP+aRT vs EBRT+BT:  2580 vs 1787 (NCDB)  RP+aRT vs EBRT+BT:  1127 vs 1149 (SEER) | NA | OS | HR (RP+aRT/EBRT+BT): 1.10 (0.95–1.27) P=0.220 (NCDB) | |
|  |  |  | CSS | HR (RP+aRT/EBRT+BT): 1.22 (0.88-1.71) P=0.234 (SEER) | |
| Berg 2019 | RP vs EBRT+BT: 12283 vs 1702 | NA | OS | HR (RP/EBRT+BT): 0.82 (0.70-0.95) P=0.008 | |
| Tilki 2019 | RP vs MaxRT: 372 vs 80  RP+aRT vs MaxRT: 88 vs 80  MaxRP vs MaxRT: 50 vs 80  RP+ADT vs MaxRT: 49 vs 80 | EBRT: 45 Gy  aRT: 68.4 Gy  BT: 108 Gy/90 Gy/100 Gy | CSS | HR (RP/MaxRT): 2.80 (1.26-6.22) P=0.01  HR (RP+aRT/MaxRT): 0.52 (0.14-1.98) P=0.34  HR (MaxRP/MaxRT): 1.33 (0.49-3.64) P=0.58  HR (RP+ADT/MaxRT): 3.15 (1.32-7.55) P=0.01 | |
|  |  |  | OS | HR (RP/MaxRT): 1.65 (0.94-2.91) P=0.08  HR (RP+aRT/MaxRT): 0.70 (0.31-1.57) P=0.39  HR (MaxRP/MaxRT): 0.80 (0.36-1.81) P=0.60  HR (RP+ADT/MaxRT): 2.33 (1.23-4.42) P=0.01 | |
| Jang 2018 | RP+aRT vs RT: 848 vs 3272 | NA | CSS | HR (RP+aRT/RT, low GS): 0.43 (0.22-0.83)  HR (RP+aRT/RT, high GS): 0.56 (0.37-0.85) | |
|  |  |  | OS | HR (RP+aRT/RT, low GS): 0.75 (0.56-1.01)  HR (RP+aRT/RT, high GS): 0.72 (0.56-0.93) | |
| Tyson 2018 | RP vs RT: 219 vs 189 | NA | QOL | Urinary | 3-yr No leakage whatsoever: RP:22% EBRT: 53% P<0.001  Urinary irritative domains: no significant difference |
|  |  |  |  | Sexual | 3-yr sexual function score: RP: 32 points EBRT: 38 points p=0.03  Erections firm enough for intercourse: RP: 22% EBRT: 18% p=0.4 |
|  |  |  |  | Bowel | No significant difference |
| Ennis 2018 | RP VS EBRT+BT: 24688 vs 2642  RP vs EBRT: 24688 vs 15435 | NA | OS | HR (RP/EBRT+BT): 0.85 (0.65-1.14)  HR (RP/EBRT): 0.65 (0.52-0.82) | |
| Gu 2018 | RP vs EBRT: 3828 vs 3828 | NA | CSS | HR (RP/EBRT): 0.552 (0.435-0.702) P<0.001 | |
|  |  |  | OS | HR (RP/EBRT): 0.675 (0.590-0.772) P<0.001 | |
| Markovina 2018 | RP vs EBRT: 62 vs 62 | 75.6 Gy | OS | HR (RP/EBRT): 0.63 (0.22-1.79) P=0.385 | |
|  |  |  | MFS | HR (RP/EBRT): 4.35 (1.41-14.29) P=0.011 | |
| Kishan 2018 | RP vs EBRT+BT: 639 vs 436  RP vs EBRT: 639 vs 734 | EBRT: 74.3 Gy  EBRT+BT: 91.5 Gy | CSS | HR (RP/EBRT+BT): 2.63 (1.47-4.76) P=0.001  HR (RP/EBRT): 1.09 (0.79-1.49) P=0.60 | |
|  |  |  | OS | ≤7.5-yr: HR (RP/EBRT+BT): 1.52 (1.04-2.17) P=0.03  HR (RP/EBRT): 0.93 (0.69-1.25) P=0.64  >7.5-yr: HR (RP/EBRT): 0.75 (0.47-1.18) P=0.21  HR (RP/EBRT+BT): 0.86 (0.52-1.43) P=0.56 | |
|  |  |  | MFS | HR (RP/EBRT+BT): 3.70 (2.33-5.88) P<0.001  HR (RP/EBRT): 1.11 (0.88-1.43) P=0.38 | |
| Robinson 2018 | RP vs RT: 3536 vs 6551 | NA | CSS | HR (RP/RT): 0.64 (0.54-0.75) | |
| Feldman 2017 | RP vs EBRT: 1429 vs 1506 | NA | CSS | HR (RP/EBRT): 0.43 (0.34-0.54) P<0.0001 | |
|  |  |  | OS | HR (RP/EBRT): 0.71 (0.55-0.92) P=0.008 | |
|  |  |  | QOL | Urinary | Urinary toxicity: RP>EBRT HR: 1.93 (1.66-2.24) |
|  |  |  |  | Sexual | Sexual toxicity: RP>EBRT HR: 5.50 (3.59-8.42) |
|  |  |  |  | Bowel | Gastrointestinal toxicity: RP<EBRT HR: 0.75 (0.65-0.86) |
| Ciezki 2017 | RP vs LDRBT: 1308 vs 515  RP vs EBRT: 1308 vs 734 | EBRT: 78 Gy/70 Gy  LDRBT: 144 Gy | CSS | HR (RP/LDRBT): 0.87 (0.45-1.67) P=0.6764  HR (RP/EBRT): 0.50 (0.32-0.77) P=0.0018 | |
|  |  |  | BRFS | HR (RP/LDRBT): 1.20 (0.95-1.52) P=0.1257  HR (RP/EBRT): 1.43 (1.19-1.79) P=0.0003 | |
|  |  |  | QOL | Urinary | 10-yr cumulative incidence of ≥grade3 genitourinary toxicity: RP: 16.4% EBRT: 8.1% LDRBT: 7.2% P<0.0001 |
|  |  |  |  | Bowel | 10-yr cumulative incidence of ≥ grade3 gastrointestinal toxicity: RP: 1% EBRT: 4.6% LDRBT: 1.1% P<0.0001 |
| Yamamoto 2015 | RP vs EBRT: 97 VS 53 | 78 Gy | QOL | Urinary | 12-mo RP vs EBRT+ADT OR: 1.99 (1.30-3.04) P=0.002  24-mo RP vs EBRT+ADT OR: 1.42 (0.95-2.11) P=0.084 |
|  |  |  |  | Sexual | 12-mo RP vs EBRT+ADT OR: 0.84 (0.62-1.14) P=0.260  24-mo RP vs EBRT+ADT OR: 0.99 (0.74-1.32) P=0.937 |
|  |  |  |  | Bowel | 12-mo RP vs EBRT+ADT OR: 0.71 (0.44-1.16) P=0.170  24-mo RP vs EBRT+ADT OR: 0.82 (0.52-1.30) P=0.400 |
| Sun 2014 | RP vs RT: 3432 vs 2152 | NA | CSS | LE <10-yr: HR (RP/RT): NA  LE≥10-yr: HR (RP/RT): 0.14 (0.03-0.60) P=0.01 | |
|  |  |  | OS | LE <10-yr: HR (RP/RT): 0.63 (0.03–11.64)  LE≥10-yr: HR (RP/RT): 0.54 (0.32–0.93) | |
| Hoffman 2013 | RP vs EBRT 381 vs 56 | NA | CSS | HR (RP/EBRT): 0.36 (0.20 -0.64) | |
|  |  |  | OS | HR (RP/EBRT): 0.65 (0.48 - 0.87) | |
| Kibel 2012 | RP vs EBRT: 525 vs 676  RP vs BT: 525 vs 33 | EBRT: 78 Gy (Clinic 1)  BT: 144 Gy (Clinic 1)  EBRT: 74 Gy (Clinic 2)  BT: 145 Gy (Clinic 2) | CSS | HR (RP/EBRT): 0.77 (0.48-1.25) P=0.2  HR (RP/BT): 0.625 (0.15-2.5) P=0.5 | |
|  |  |  | OS | HR (RP/EBRT): 0.59 (0.43-0.77) P=0.001  HR (RP/BT): 0.32 (0.17-0.59) P<0.001 | |
| Westover 2012 | RP vs EBRT+BT: 285 vs 372 | EBRT:45 Gy  BT:108 Gy/90 Gy/100 Gy | CSS | HR (RP/EBRT+BT): 1.8 (0.6 – 5.5) P=0.3 | |
| Boorjian 2011 | RP vs EBRT+ADT: 1238 vs 344  RP vs EBRT: 1238 vs 265 | 72 Gy | CSS | HR (RP/EBRT+ADT): 0.88 (0.52-1.47) P=0.61  HR (RP/EBRT): 0.47 (0.29-0.74) P=0.001 | |
|  |  |  | OS | HR (RP/EBRT+ADT): 0.625 (0.49-0.8) P=0.0002  HR (RP/EBRT): 0.49 (0.39-0.62) P<0.0001 | |
| Aizer 2009 | RP vs EBRT: 42 vs 117(MSK) | 75.6 Gy | BRFS | HR (RP/EBRT): 2.56 (1.30-5) P=0.006 (MSK) | |
| Takizawa 2009 | RP vs EBRT: 24 vs 37 | 70-71 Gy | QOL | Urinary: | Urinary function (UCLA PCI scores): RP: 85.7±30.7 EBRT:93.9±14.7 P=0.050  Urinary bother: RP: 89.6±22.5 EBRT: 96.9±8.4 P=0.298 |
|  |  |  |  | Sexual | Sexual function (UCLA PCI scores): RP:4.0 ±10.2 EBRT: 2.2±21.3 P<0.001  Sexual bother: RP: 73.1±34.6 EBRT: 80.4±21.3 P=0.433 |
|  |  |  |  | Bowel | Bowel function (UCLA PCI scores): RP: 90.2 ±21.5 EBRT: 92.9±9.2 P=0.443  Bowel bother: RP: 88.5±24.2 EBRT: 95.0±12.5 P=0.375 |
| Arcangeli 2009 | RP vs EBRT: 122 vs 162 | 80 Gy/62 Gy | BRFS | HR (RP/EBRT): 2.47 (1.37-4.45) p=0.002 | |
| Akakura 2006 | RP vs EBRT: 46 vs 49 | 60-70 Gy | QOL | Urinary: | Incontinence: RP>RT(P<0.01) |
|  |  |  |  | Sexual | No significant difference |
|  |  |  |  | Bowel | No significant difference |

RP: prostatectomy; RT: radiotherapy; EBRT: external beam radiation therapy; BT: brachytherapy; LDRBT: low dose rate brachytherapy; ADT: androgen deprivation therapy; aRT: adjuvant radiotherapy; WW: watchful waiting; MaxRP: RP+aRT+ADT; MaxRT: EBRT+BT+ADT; MSK: Memorial Sloan Kettering; OS: overall survival; CSS: cancer specific survival; BRFS: biochemical recurrence free survival; MFS: metastasis free survival; CRF: clinical recurrence free survival; HR: hazard ratio; OR: odds ratio; PSA: prostate-specific antigen; GS: Gleason Score; CL: confidence interval; IV: inverse variance; SE: standard error; vs: versus; NA: not available; UCLA PCI scores: the University of California Los Angeles Prostate Cancer Index scores. NCDB: The National Cancer Data Base; SEER: Surveillance; Epidemiology; and End Results.
