# Supplementary material for: The efficacy and safety of radical prostatectomy and radiotherapy in high-risk prostate cancer: a systematic review and meta-analysis
Source: World J Surg Oncol. 2020 Feb 24;18:42. doi: 10.1186/s12957-020-01824-9 (PMC7041271; doi:10.1186/s12957-020-01824-9)
Supplement: Supplementary file 2 — Additional file 2: Table S2. Quality assessment of the included studies. [file 12957_2020_1824_MOESM2_ESM.docx]

**Supplementary Table 2. Quality assessment of the included studies**

| Study ID | Selection | Comparability | Outcome | Total |
| --- | --- | --- | --- | --- |
| Jayadevappa 2019 | 4 | 1 | 3 | 8 |
| Reichard 2019 | 4 | 1 | 3 | 8 |
| Caño-Velasco 2019 | 4 | 1 | 3 | 9 |
| Muralidhar 2019 | 4 | 0 | 3 | 7 |
| Berg 2019 | 4 | 0 | 3 | 7 |
| Tilki 2019 | 4 | 2 | 3 | 9 |
| Jang 2018 | 4 | 2 | 3 | 9 |
| Tyson 2018 | 4 | 0 | 3 | 7 |
| Ennis 2018 | 4 | 2 | 3 | 9 |
| Gu 2018 | 4 | 2 | 3 | 9 |
| Markovina 2018 | 4 | 2 | 3 | 9 |
| Kishan 2018 | 4 | 2 | 3 | 9 |
| Robinson 2018 | 4 | 0 | 3 | 7 |
| Feldman 2017 | 4 | 2 | 3 | 8 |
| Ciezki 2017 | 4 | 0 | 3 | 7 |
| Yamamoto 2015 | 4 | 0 | 2 | 6 |
| Sun 2014 | 4 | 0 | 3 | 7 |
| Hoffman 2013 | 4 | 0 | 3 | 8 |
| Kibel 2012 | 4 | 2 | 3 | 9 |
| Westover 2012 | 4 | 0 | 3 | 7 |
| Boorjian 2011 | 4 | 0 | 3 | 7 |
| Aizer 2009 | 4 | 0 | 3 | 7 |
| Takizawa 2009 | 4 | 0 | 3 | 7 |
| Arcangeli 2009 | 4 | 0 | 2 | 6 |
