# Supplementary figures and images for: The efficacy and safety of radical prostatectomy and radiotherapy in high-risk prostate cancer: a systematic review and meta-analysis
Source: World J Surg Oncol. 2020 Feb 24;18:42. doi: 10.1186/s12957-020-01824-9 (PMC7041271; doi:10.1186/s12957-020-01824-9)

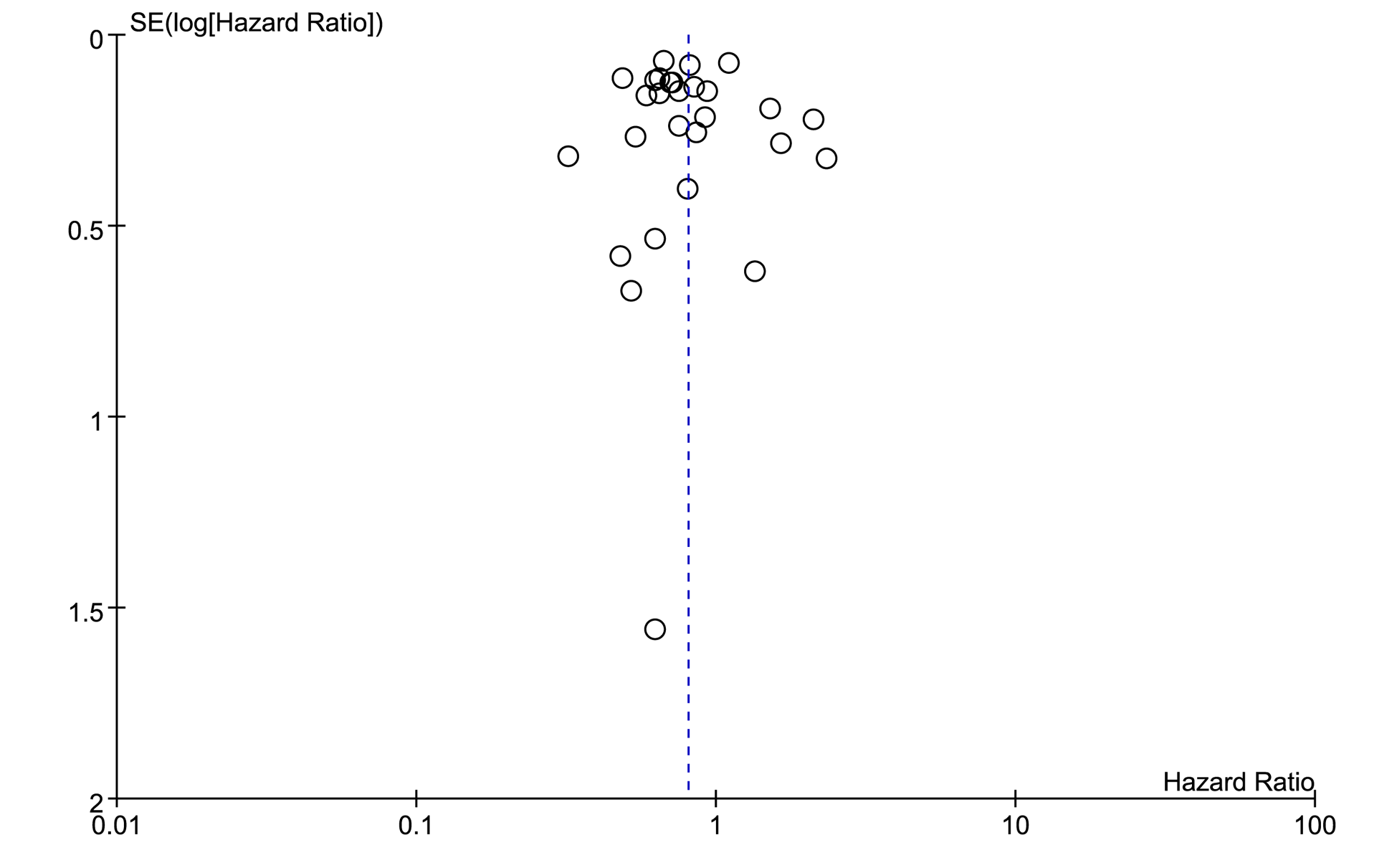

Supplement: Supplementary file 3 — Additional file 3: Figure S1. Funnel plot of radical prostatectomy versus radiotherapy using outcome of overall survival. [file 12957_2020_1824_MOESM3_ESM.tif]
